# Supplementary material for: Chromothripsis during telomere crisis is independent of NHEJ, and consistent with a replicative origin
Source: Genome Res. 2019 May;29(5):737–49. doi: 10.1101/gr.240705.118 (PMC6499312; doi:10.1101/gr.240705.118)
Supplement: Supplemental Material [file supp_gr.240705.118_Supplemental_file_1.zip › contigs/annotated_contigs/DB93/contig.2.DB93_length_548_mean_cov_4.92700729927.docx]

**DB93_length_548_mean_cov_4.92700729927**

```
CGTCTGGCTCTTTCTGCATTTGATAAAGTGACTGACGGATGCTAGGTGCTCAATAAACATCTGTTAAGTTAACTCTGACTTTCTCGTGG
 >chr19:29357461-29357786 + E=5e-184 p=6e-03
TTGGAACAGCTATCATCAGAGCACACCAAGAGACAGGTTTAGGAGCCTCCATCCTCTTGTATACGTAAGAGGATCTGAGTGGAGGTTCC

CCAGAAGCAGATCTGGAGATGAAGATTTGAGGGCAAGTAGTTTATTTAGATGAAATTAGGAAGAAAAGGGAAAACCACCAGTCAGGTGC

AGCTATGGAGCCAGCCACCACCATGGGCGGGTGGAGCTGAATCCCGTGGGGAACTCT|G|ATACCAATTACAAGTAGTAAGTTGCCATC
                                                          >chr19:29356901-29357125 - E=
TATGCGTCTGACCCATCAGCTGTAAGTTGGGATTCCCACCACTCCCTTGGGTTTGCTTAATTTGCTAGGATGGCTCACAGAACTCACGG
4e-123
AAGCATGTTTACCATTTTATTATAAAGGATGTCATCATGGACACAGATGGACAGCCAGAGGAGGAGAGTCACAAGGTAAGGTTTGGGGT

GGAGCCTCCATGCCCT
```
